# CheNER: a tool for the identification of chemical entities and their classes in biomedical literature

**DOI:** 10.1186/1758-2946-7-S1-S15

**Published:** 2015-01-19

**Authors:** Anabel Usié, Joaquim Cruz, Jorge Comas, Francesc Solsona, Rui Alves

**Affiliations:** 1 Departament Ciències Mèdiques Bàsiques, Universitat de Lleida, Av. Rovira Roure nº 80, 25298 Lleida, Spain; 2 Departament d'Informàtica i Enginyeria Industrial, Universitat de Lleida, C/Jaume II nº 69, 25001, Lleida, Spain; 3 Centro de Biotecnologia Agricola e Agro-Alimentar do Baixo Alentejo (CEBAL), Rua. Pedro Soares s/n, Campus IPBeja, 6158 7801-908 Beja, Portugal

## Abstract

**Background:**

Small chemical molecules regulate biological processes at the molecular level. Those molecules are often involved in causing or treating pathological states. Automatically identifying such molecules in biomedical text is difficult due to both, the diverse morphology of chemical names and the alternative types of nomenclature that are simultaneously used to describe them. To address these issues, the last BioCreAtIvE challenge proposed a CHEMDNER task, which is a Named Entity Recognition (NER) challenge that aims at labelling different types of chemical names in biomedical text.

**Methods:**

To address this challenge we tested various approaches to recognizing chemical entities in biomedical documents. These approaches range from linear Conditional Random Fields (CRFs) to a combination of CRFs with regular expression and dictionary matching, followed by a post-processing step to tag those chemical names in a corpus of Medline abstracts. We named our best performing systems CheNER.

**Results:**

We evaluate the performance of the various approaches using the F-score statistics. Higher F-scores indicate better performance. The highest F-score we obtain in identifying unique chemical entities is 72.88%. The highest F-score we obtain in identifying all chemical entities is 73.07%. We also evaluate the F-Score of combining our system with ChemSpot, and find an increase from 72.88% to 73.83%.

**Conclusions:**

CheNER presents a valid alternative for automated annotation of chemical entities in biomedical documents. In addition, CheNER may be used to derive new features to train newer methods for tagging chemical entities. CheNER can be downloaded from http://metres.udl.cat and included in text annotation pipelines.

## Background

Scientific literature accumulates at a rate that makes it impossible for any biologist to extract all the relevant information from the multitude of available sources. For this reason, there is a keen interest in the development of systems that can automatically mine information from the text and provide that information to researchers.

Mining biologically important information from text is a two-step process, requiring that one identifies the relevant entities in the documents and, subsequently, the relationships between those entities. Methods that fully automate both steps of the process in a combined way with highly accurate results have yet to be developed. So far the focus has been mostly on creating and testing methods that perform one of the steps of the text-mining process (see for example [1-8]). This focus has been further promoted by initiatives such as the BioCreAtIvE challenge (BioCreAtIvE Workshops I, II, II.5, III, and IV held in 2004, 2007, 2009, 2010, and 2013 respectively) [1-5].

The BioCreAtIvE challenge provides participating research teams with annotated literature corpora that enable a controlled comparison of the performance between the various competing methods for automated recognition of specific types of entities in biomedical documents. There are various BioCreAtIvE challenge tracks that focus on identifying various types of biologically relevant entities, such as genes and their functions, diseases, phenotypes, or chemical compounds. The importance of these chemical compounds arises from their involvement in regulating biological activity of proteins and genes, and from their potential use to treat pathological states.

Identifying chemical entities in biomedical textbooks, patents, articles, and other scientific documents is a challenging task. The difficulty arises from two main factors: the diverse morphology of chemical entities and the various types of nomenclature that are simultaneously used to describe them in biomedical documents [9]. These factors make it difficult to develop a single approach that can successfully identify all types of chemical mentions with high accuracy. Because of this there is a small number of applications available to do NER of chemical names [10-22]. In addition, many of these applications are not freely available to the community, as summarized in Table 1.

**Table 1:** Examples of chemical entity recognition applications.

Applications	Availability
ProMiner [10]	CL
Whatizit [11]	F
Chemical Reader (MDL and TEMIS) [12]	CL
Oscar3/4 [13,14]	F
K&K CRF [15,16]	NA
ChemicalTagger [17]	F
SureChem [18]	CL (TVA)
ChemFinder (ChemBioFinder) [19]	CL (TVA)
Chemical Name Spotter UIMA,IBM [20]	CL
ChemSpot[21]	F
CheNER[22]	F

CL: Commercial License, NA: Not Available, F: Free, TVA: Trial Version Available

Some of the most accurate approaches for the automated identification of chemical entities use Conditional Random Fields (CRFs) [15,16,21,22], Maximum Entropy Markov Models (MEMM) [13,14], or Support Vector Machines (SVM) [23]. These approaches employ statistical methods to identify chemical entities. Often, the performance of statistical methods can be improved by combining them with linguistic analysis techniques [24-27]. A detailed review about this subject can be found in [9].

The statistical methods used to identify chemical entities must be trained through the use of appropriate and encompassing gold standard collections of documents (corpora), containing precisely annotated chemical entities [5]. Although quite useful, existing corpora [15,16,28,29] that can be used for training those methods are often limiting in developing automatic annotation systems, because they are small in size and have incomplete annotation. The DDI corpora contain a larger number of documents (766) and chemical entities (13029). However, it is only adequate to train methods that perform NER of pharmacological substances. Because of this only the SCAI corpora could be considered as a general gold standard that covered a large class of chemical entities, containing a total number of ~1550 abstracts with ~6600 entities annotated. However, the Medline corpus within the SCAI corpora only contains 100 Medline abstract with 151 annotated IUPAC (International Union of Pure and Applied Chemistry) chemical names.

The latest round of the BioCreAtIvE challenge emphasized how important automated annotation of chemical entities in biomedical documents is by setting up a track (CHEMDNER) to potentiate the development of more accurate methods to perform that annotation. In order to lift one the main limitations in developing annotation methods, two new biological literature corpora with annotated chemical entities were provided for the community to use in training their methods. Each corpus contains 3500 documents, with approximately 29500 annotated chemical entities, divided into several classes: SYSTEMATIC, TRIVIAL, FAMILY, FORMULA, ABBREVIATIONS, IDENTIFIERS, MULTIPLE, and NO CLASS. The corpora developed by BioCreAtIvE IV are significantly larger than the SCAI corpora [15,16] and the DDI corpora [28,29] that were freely available for the training and testing of applications that perform chemical NER. Our team had previously developed CheNER, a tool that automatically and specifically tags IUPAC chemical names in documents [22]. CheNER uses CRFs based on Mallet [30] to identify the IUPAC names and achieves F-score performances higher than 70% in the SCAI corpora [15,16]. Given that the IUPAC nomenclature is only one of the many that are used, we took the opportunity provided by BioCreAtIvE IV organizers to further develop CheNER in order for it to specifically identify and tag the different classes of chemical names.

In this paper we report the development of this improved version of CheNER and analyse its performance. We implemented and tested a set of approaches that combine dictionary matching, linear CRFs and regular expressions in different ways to tag chemical entities according to their nomenclature classes in the biomedical literature. We find that the approach with the highest performance implements a CRF that is trained to simultaneously identify the individual classes of chemical entities. Our system is freely available at http://metres.udl.cat and can be easily integrated in pipelines to annotate large bodies of literature. To our knowledge, CheNER is unique with respect to other chemical entity annotation programs that were presented during the challenge because CheNER groups the chemical terms it annotates into the various classes of chemical names.

## Materials & methods

Our set of approaches combines CRFs, dictionary matching, and regular expression matching in five different ways (Table 2; also see below for details). We defined two different taggers: CRFs tagger and Regular Expression tagger (which include dictionary and regular expression approaches).

**Table 2:** Sets of approaches combining CRFs, dictionary matching, and regular expression matching in five different ways.

Run	Description
1	Combines a CRF to identify SYSTEMATIC entities with dictionary matching to identify TRIVIAL, FAMILY, and ABBREVIATION entities, and regular expression matching to identify FORMULA and IDENTIFIER entities.
2	Combines individual CRFs to identify SYSTEMATIC and TRIVIAL entities with dictionary matching to identify FAMILY and ABBREVIATION entities, and regular expression matching to identify FORMULA and IDENTIFIER entities.
3	Uses a single CRF to identify SYSTEMATIC, TRIVIAL, FAMILY, ABBREVIATION, FORMULA and IDENTIFIER entities.
4	Combines individual CRFs to identify SYSTEMATIC, TRIVIAL, FAMILY, ABBREVIATION, and FORMULA entities with an individual regular expression matching to identify IDENTIFIER entities.
5	Uses a single CRF to identify SYSTEMATIC, TRIVIAL, FAMILY, ABBREVIATION, FORMULA and IDENTIFIER entities and specifically labels each class of entity.

### CRF implementation

In the original development of CheNER we systematically tested how order, offset conjunction, and tokenization affected the performance of the CRF [22]. Based on those tests we decided to use linear chain, 2^nd ^order CRFs, with an offset conjunction value of 1 and tokenization by spaces in the development of the current CheNER version. We note that the punctuation marks at the end of the tokens are not taken it into account to extract their features. All CRFs for the current work were implemented using Mallet [30], and trained using the training corpus provided by the BioCreAtIvE organizers, containing 3500 abstracts, with ~29500 annotated entities.

### Word features, regular expressions, and dictionaries

The features used to originally train CheNER's CRF [22] were also used in the current work. However, we note that the first version of CheNER was developed to specifically identify IUPAC chemical names. The BioCreAtIvE IV CHEMDNER track that CheNER participated in called for identifying and annotating all types of chemical entities. In order to accommodate for this we added the features described in Table 3 to the training process. These features were chosen because they have been previously identified as the best subset of features that better discriminates chemical names [15,16].

**Table 3:** Examples of features and regular expressions used during the training of the chemical entities identification systems.

Name of feature	Description
Length	Classifies tokens by length. If the length is less than 5, the token is Short. If length is between 5 and 15, the token is Medium, otherwise, the token is Large.

Word class	Automatic generation of features in terms of frequency of upper and lower case characters, digits and other types of characters.

Autom. Prefixes/Suffixes	Automatic generation of suffix and prefix (length 2, 3 and 4)

List	Automatic generation for every token that match an element within the list. We used lists of basic name segments (~3300), and stop words (~550).

Dictionaries	A dictionary matching for trivial, family and abbreviations names classes (~6400, ~1300 and ~1400 elements, repectively).

Regular expressions	Regular expressions that identify specific features, such as "contains dashes?", "is all cap?", or "contains numbers?". Regular expressions that identify specific types of characters that are more common in chemical entities than in other words, such as greek letters, roman numbers, etc. Regular expressions that match with specific morphological chemical formulas features, identifiers, and systematic features in chemical names. Regular expressions used in the pos-processing step that filter out common names that are incorrectly tagged by the systems in a systematic way.

Given that several classes of chemical names present either a very regular structure or a finite set of names, we wanted to see if using regular expressions and/or dictionaries to identify the entities for those classes would perform as well as using CRFs. The classes for which we wanted to test this were TRIVIAL, FAMILY, ABBREVIATION, FORMULA, and IDENTIFIER chemical names. The regular expressions that were defined to train our system in the runs that combine CRFs and Regular Expression taggers are also summarized in Table 3. FORMULA chemical were identified in these runs by using regular expressions describing patterns containing atomic elements, SMILES, etc. The dictionaries used to identify TRIVIAL, FAMILY, and ABBREVIATIONS in the relevant runs were built from a non-redundant list of the entities from each class annotated in the corpora provided by the BioCreAtIvE organizers, the SCAI corpora, and also by extracting the names of chemical entities from http://www.drugs.com/. In total, these dictionaries have ~9100 terms, with ~6400 for the TRIVIAL dictionary, ~1300 for the ABBREVIATION dictionary and ~1400 for the FAMILY dictionary. To identify SYSTEMATIC names using a CRF, we used regular expressions to define patterns that identify morphological structures such as isomers (ex: 3,5,4'-trihydroxy-trans-stilbene), as well as the expressions used in [22]. We note that regular expressions or dictionary words used to identify any type of chemical entity by the Regular Expression tagger were also used as a feature to identify the same type of entities by the CRFs tagger in the relevant runs.

It is likely that overall performance of our system would improve by including additional dictionaries such as ChEBI [31,32], Jochem [33] and PubChem [34]. However, the deadlines of the BioCreAtIvE challenge made it impossible to develop a reasonable way to correctly attribute class type to each entity in these dictionaries, and class attribution was a differential feature that we wanted CheNER to have.

#### Runs

We tested five different approaches (Runs) to Chemical NER, in order to see which approach works better in the global identification of the chemical names. Each of these Runs is described in Table 2.

#### Output

The output of the CRFs, dictionary, and Regular Expression taggers in each run is marked according to the IOB (In-Out-Beginning) labelling scheme [9]. This output is reformatted to the required specifications of the CDI (Chemical Document Indexing) and/or CEM (Chemical Entity Mention) output format.

**Figure 1:**
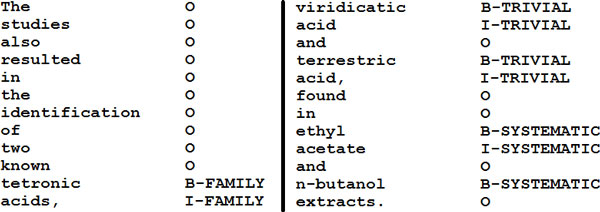
**Example of how chemical entity class names are tagged by CheNER using the IOB scheme format**. Tokens that are not recognized as chemical entities are marked with O. Tokens that are recognized as the beginning of a chemical entity are marked with B. Tokens that are recognized as continuing the name of a chemical entity are marked with I. In addition, CheNER adds the class of the chemical name it tags.

The integration of the output from the various recognition approaches used in a run (CRF, dictionary, and regular expression matching) is done through a post-processing step. In this step we perform several clean up actions, such as correcting unequal numbers of closing or opening brackets or detagging "action words" that are often appended at the end of chemical mentions such as "-based", "-regulated", etc. This clean up is done in the following way. Once the names are tagged by all the approaches, the systems remove all the mention that match with regular expressions that eliminate various classes of potential False Positive entities detected. In addition, regular expression matching is also used to correct the mentions that contain "action words". Once this clean up is done, the output of all approaches is merged and tagged using the IOB scheme (see Figure 1 for examples).

### Evaluation of the results

The F-score is a standard way to evaluate performance of NER methods [9]. It is given by the harmonic mean between precision and recall. We calculate the micro-averaged F-score of the individual Runs over the development and test corpora, which is the evaluation measure used by the BioCreAtIvE IV organizers. The micro-averaged performance is calculated by weighing equally every annotated entity in the corpus. To get the macro-averaged scores, each document should be evaluated, and then the resulting evaluation should be averaged on the whole corpus. The calculations of precision, recall, and F-score are done using the evaluation library provided by the BioCreAtIvE IV organizers, downloaded from http://www.biocreative.org/resources/biocreative-ii5/evaluation-library/.

## Results & discussion

The evaluation of the systems presented to the IV BioCreAtIvE workshop was done by the organizers using a subset of 3000 abstracts within a test data set composed of 20000 abstracts, and calculating micro-averaged precision, recall, and balanced F-score. The performance of the systems was calculated with the BioCreAtIvE evaluation library.

### Performance of the five runs

The performance of the systems implemented in each run was tested using the CHEMDNER development corpus in two different ways. On one hand we tested the performance of the system in identifying unique chemical entities in the documents of the corpus (CDI subtask). Table 4 summarizes the results and we see that the system implemented in Run 5 has the highest F-Score performance. On the other hand, we tested the performance of each system in identifying all mentions of chemical entities in the documents of the corpus (CEM subtask). Table 5 summarizes the results and again, we see that the system implemented in Run 5 has the highest F-Score performance. In addition, we see that the system implemented in Run 5 has similar performance in the two tasks, suggesting that it might be at the higher limit of performance for the set of features considered during the training of the CRFs. We remind readers that the system implemented in Run 5 uses a single CRF that simultaneously identifies both, chemical entities and their classes.

**Table 4:** Micro-average CDI subtask results.

	Run 1	Run 2	Run 3	Run 4	Run5
**P**	77.37	80.79	83.01	83.17	76.79
**R**	65.58	56.44	54.79	61.36	69.36
**F**	70.99	66.45	66.01	70.62	72.88
**AP**	50.25	44.83	44.94	50.70	52.18
**Fs**	58.85	53.54	53.48	59.02	60.82

P:precision; R:recall; F:F-score; AP: average precision; Fs: harmonic mean between AP and F-score.

**Table 5:** Micro-average CEM subtask results.

	Run 1	Run 2	Run 3	Run 4	Run5
**P**	77.58	80.49	85.17	85.15	81.49
**R**	65.71	66.13	48.72	59.45	66.23
**F**	71.15	72.61	61.98	70.02	73.07
**AP**	49.79	50.35	40.13	49.23	51.82
**Fs**	58.58	49.47	48.71	57.85	60.64

P: precision; R: recall; F: F-score; AP: average precision; Fs: harmonic mean between AP and F-score.

What causes the differences in performance between the various approaches we use to identify chemical entities? For example, the approach in Run 3 has the lowest F-score in both subtask, CDI and CEM. This run implements an individual CRF for each entity class. The CRF that identifies FORMULA chemical names tags a large number of false positives, leading to a very low recall. This is seen by comparing the results from Run 3 and Run 4. These two runs differ only in how the system identifies the FORMULA chemical names. We see that the identification of FORMULA chemical names using a single CRF decreases the recall by ~15% when compared to FORMULA identification using regular expressions. This suggests that the context where FORMULA names are often found in the text is not sufficiently informative to allow the CRF to appropriately rule out many false positives.

We see a similar effect in Run 2. This Run has an F-score closer to Run 3 in the CDI subtask, while its F-score in the CEM task is closer to that of the best system. This difference is due to the fact that the system missed more unique entities than systems using CRFs to identify FAMILY, ABBREVIATION, FORMULA and IDENTIFIER chemical names. However, the entities of these types identified by Run 2 are the most frequently repeated in the texts that are analyzed, which raises the F-score of this Run in the CEM task.

To summarize, the usage of a single CRF for each entity class leads to many false positives for each class, due to the similitude between the entity types. Replacing some CRFs with the direct use of Regular Expression taggers leads to a smaller number of entities being identified but improves the identification of the class for those entities, decreasing false positives. When a single CRF is used to tag all classes of entities (Run 5), this CRF can create a more accurate model for each class, thus improving the ability of the method to clearly identify the difference between the entity classes.

In the evaluation done for the BioCreAtIvE Challenge, the best system presented by CheNER achieves an F-score of 67.78 % in the CDI task and an F-score of 63.74% in the CEM task. These scores are higher in the development corpus (72.08% F-score in the CDI task and 72.61% F-score in the CEM task). The version of CheNER we present in this work improves the original F-scores from the BioCreAtIvE workshop to 72.68% in the CDI task and 73.07% in the CEM task. This increase in F-Score indicates that the new version of CheNER has an improved performance. Nevertheless, it would be important to calculate the performances for both tasks once the ***annotated ***test corpus becomes available to make sure that performance has also improved in that corpus.

### Merging the tagging results from different chemical NER tools

The systems with the highest F-score performance in the BioCreAtIvE challenge were trained by combining features that are derived from a human analysis of patterns in chemical names to features that are derived from the automated tagging of chemical entities by entities such as OSCAR or ChemSpot [35-44]. All these systems have F-scores that are 10%-15% higher than those of CheNER, which uses only human-derived features.

We wanted to see whether adding features derived from the automated tagging by CheNER to those combined systems could improve their performance. These features would, for example, be the annotated chemical names themselves. To test this directly we would have to include the output of CheNER ourselves into the tools described in [35-44] and measure the resulting F-Score. However, the relevant tools were not publicly available and this conclusive experiment could not be performed.

As an alternative test to see whether adding features derived from the automated tagging by CheNER to those combined systems might improve their performance, we merged the individual results of CheNER [22], OSCAR [13,14], and ChemSpot [21] in tagging the CHEMDNER development corpus. This allowed us to investigate whether the three programs identified largely overlapping sets of entities or not. We did this for the CDI subtask.

The experiment was done in the following way. Each of the three tools was run in the CHEMDNER development corpus. The entities tagged by each tool were then filtered through the post-processing step described in Methods for CheNER. After post-processing, the precision, recall, and F-Score were recalculated for the combinations of CheNER, OSCAR, and ChemSpot described in Table 6. We find that the performance of OSCAR and ChemSpot improves by a few percent when the post-processing step we developed is applied to the entities that they tag. However, this improvement is not enough to compensate for the low precision achieved by OSCAR.

**Table 6:** Comparative micro-average performance evaluation of "out of the box" versions of ChemSpot and OSCAR.

	NO processing of results			Processing of results		
	**P**	**R**	**F**	**P**	**R**	**F**

**ChemSpot**	70.05	59.63	64.43	71.86	59.81	65.28
**OSCAR**	29.97	79.95	43.60	35.26	80.00	48.95

P: precision; R: recall; F: F-score. No processing: results were not processed through the post-processing step described in methods; Processing of results: results were passed through the post-processing step described in methods.

If we compare Tables 4 and 6, we see that CheNER always outperforms the other two programs, when they are run in their "out of the box" version, meaning that the tool can be downloaded from the Internet http://metres.udl.cat/ and used as is in annotation pipelines. In addition, Table 7 shows that combining CheNER and ChemSpot improves the individual performance of either tool. However, combining both tools with OSCAR significantly decreases the F-Score with respect to either CheNER or OSCAR. This is a consequence of the low precision shown by OSCAR.

**Table 7:** Comparative F-Score performance combining "out of the box" versions of ChemSpot, OSCAR, and CheNER.

	Run 1	Run 2	Run 3	Run 4	Run5
**CheNER**	70.99	66.45	66.01	70.62	72.88
**CheNER+ChemSpot**	73.05	70.03	73.31	73.83	73.18
**CheNER+ChemSpot+OSCAR**	50.28	50.31	50.86	50.81	50.10

Overall, our results show that combining the result list of CheNER and ChemSpot improves the performance of either tool (Tables 4, 6, 7). We find that there are 2643 annotated chemical entities that are only recognized by ChemSpot and 2893 annotated chemical entities that are only recognized by CheNER (Table 8). Taken together, the results from Tables 4, 5, 6, 7, 8 suggest that including CheNER in combination with ChemSpot could improve the performance of methods that combine several tools.

**Table 8:** Comparative analysis of true and false positive tagging between the best run of CheNER and ChemSpot.

	True Positives	False Positives	Unique True Positives	Unique False Positives
ChemSpot	9626	3769	2643	3297
CheNER	9876	1999	2893	1527

### Notes on the IV BioCreAtIvE Challenge

**Figure 2:**
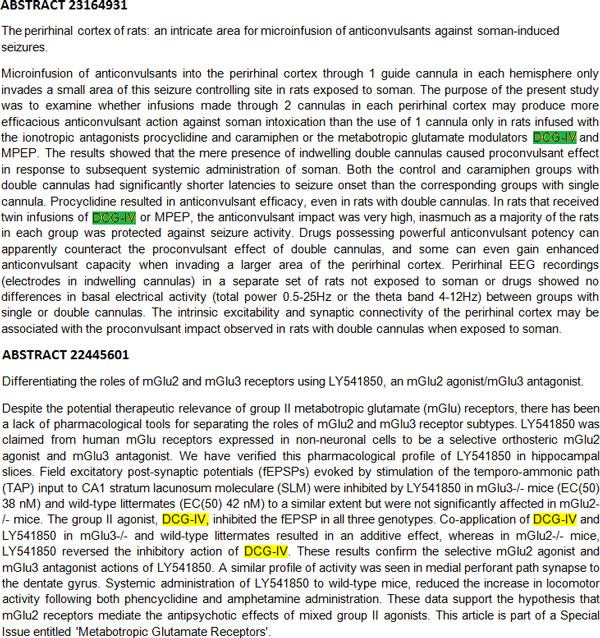
**Example of an entity that is not consistently annotated over different abstracts**. DCG-IV is correctly annotated as a chemical entity in Abstract 23164931. However, it is not annotated at all in Abstract 22445601.

One of the most important outcomes from the BioCreAtIvE IV Challenge is the development of larger sized literature corpora that can be used for the training and evaluation of automated chemical entity annotation systems. Specifically, two corpora of 3500 abstracts each for training and development, and a test corpus containing more than 20000 abstracts are invaluable resources for the development of better chemical annotation systems. However, even these corpora should be further curated and, to some extent, reannotated. This is so because there is small percentage of cases where the same chemical entities were either not consistently annotated over different abstracts or not recognized as chemical entities by the annotators (see Figure 2 for examples). In addition, there are still some problems with the normalization of chemical entity names in documents. The methods presented in this volume could highly facilitate this process if a semi-automated reannotation approach is applied.

## Conclusions

Here we presented CheNER, the latest version of our system for chemical entity tagging in biological literature. While the original version of CheNER only tagged IUPAC names, the current version tags and identifies various classes of chemical entities (see Figure 1 for an example), with a performance that is better than that of other comparable tools that can be downloaded from the internet and used "out of the box" (see Tables 4, 6, and 7 and references [5] and [35]). This version is a development over the one we presented at the IV BioCreAtIvE Challenge workshop, where we only presented early results from Runs 1, 2, 4 in the CDI subtask and Run 1 in the CEM subtask [5]. In addition to testing additional systems, we further refined the post-processing of the results, significantly improving our F-Score.

CheNER presents a valid alternative for automated annotation of chemical entities in biomedical documents that can be downloaded from http://metres.udl.cat and easily integrated in annotation workflows. Examples on how to perform this integration are provided in the website. The individual performance of CheNER could be further improved by expanding the dictionaries of chemical entities used in its training. In addition, CheNER may provide a valuable resource to automatically derive new features that could be used for training and improving the performance of newer methods for tagging chemical entities.

## Competing interests

The authors declare that they have no competing interests.

## Authors' contributions

AU, RA and FS conceived the study and planned the development of the tool. AU, JCz, JCs, and RA developed the regular expressions and dictionaries that are integrated in the tool. AU wrote the tool. AU carried out the computational experiments, with some assistance from RA. AU, RA, and FS analysed the results. AU, RA and FS drafted the manuscript. All authors read and approved the final manuscript.

## Declarations

Funding for publication of this article comes from grants BFU2010-17704 and TIN2011-28689-C02-02 from the Spanish Ministry of Economy and Competitiveness.

This article has been published as part of *Journal of Cheminformatics *Volume 7 Supplement 1, 2015: Text mining for chemistry and the CHEMDNER track. The full contents of the supplement are available online at http://www.jcheminf.com/supplements/7/S1.

## References

[B1] Hirschman L Yeh A Blaschke C Valencia A Overview of BioCreAtIvE: critical assessment of information extraction for biology BMC Bioinformatics 2005 6 S1 15960821 10.1186/1471-2105-6-S1-S1 PMC1869002

[B2] Krallinger M Morgan A Smith L Leitner F Tanabe L Wilbur J Hirschman L Valencia A Evaluation of text-mining systems for biology: overview of the Second BioCreative community challenge Genome Biol 2008 9 S1 18834487 10.1186/gb-2008-9-s2-s1 PMC2559980

[B3] Leitner F Mardis SA Krallinger M Cesareni G Hirschman LA Valencia A An Overview of BioCreative II.5 IEEEACM Trans Comput Biol Bioinforma IEEE ACM 2010 7 385 399 10.1109/tcbb.2010.61 20704011

[B4] Arighi C Lu Z Krallinger M Cohen K Wilbur W Valencia A Hirschman L Wu C Overview of the BioCreative III Workshop BMC Bioinformatics 2011 12 S1 22151647 10.1186/1471-2105-12-S8-S1 PMC3269932

[B5] Krallinger M Leitner F Rabal O Vazquez M Oyarzabal J Valencia A CHEMDNER: The drugs and chemical names extraction challenge J Cheminform 2015 7 Suppl 1 S1 10.1186/1758-2946-7-S1-S1 PMC4331685 25810766

[B6] Kim J-D Ohta T Pyysalo S Kano Y Tsujii J Overview of BioNLP'09 shared task on event extraction Proc Work Curr Trends Biomed Nat Lang Process Shar Task 1 9

[B7] Kim J-D Pyysalo S Ohta T Bossy R Nguyen N Tsujii J Overview of BioNLP Shared Task 2011 Proc BioNLP Shar Task 2011 Work 2011 Portland, Oregon, USA: Association for Computational Linguistics 1 6

[B8] Nédellec C Bossy R Kim J-D Kim J Ohta T Pyysalo S Zweigenbaum P Overview of BioNLP Shared Task 2013 Proc BioNLP Shar Task 2013 Work 2013 Sofia, Bugaria: Association for Computational Linguistics 1 7

[B9] Vazquez M Krallinger M Leitner F Valencia A Text Mining for Drugs and Chemical Compounds: Methods, Tools and Applications Mol Informatics 2011 30 506 519 10.1002/minf.201100005 27467152

[B10] Hanisch D Fundel K Mevissen H-T Zimmer R Fluck J ProMiner: rule-based protein and gene entity recognition BMC Bioinformatics 2005 6 S14 15960826 10.1186/1471-2105-6-S1-S14 PMC1869006

[B11] Rebholz-Schuhmann D Arregui M Gaudan S Kirsch H Jimeno A Text processing through Web services: calling Whatizit Bioinformatics 2008 24 296 298 10.1093/bioinformatics/btm557 18006544

[B12] Cooke-Fox DI Kirby GH Lord MR Rayner JD Computer translation of IUPAC systematic organic chemical nomenclature. 4. Concise connection tables to structure diagrams J Chem Inf Comput Sci 1990 30 122 127 10.1021/ci00066a004

[B13] Corbett P Murray-Rust P R Berthold M, Glen RC, Fischer I High-Throughput Identification of Chemistry in Life Science Texts Comput Life Sci II 2006 4216 Berlin, Heidelberg: Springer Berlin Heidelberg 107 118

[B14] Jessop D Adams S Willighagen E Hawizy L Murray-Rust P OSCAR4: a flexible architecture for chemical text-mining J Cheminformatics 2011 3 41 10.1186/1758-2946-3-41 PMC3205045 21999457

[B15] Klinger R Kolářik C Fluck J Hofmann-Apitius M Friedrich CM Detection of IUPAC and IUPAC-like chemical names Bioinformatics 2008 24 i268 i276 10.1093/bioinformatics/btn181 18586724 PMC2718657

[B16] Kolářik C Klinger R Friedrich CM Hofmann-apitius M Fluck J Chemical Names: Terminological Resources and Corpora Annotation 2008

[B17] Hawizy L Jessop D Adams N Murray-Rust P ChemicalTagger: A tool for semantic text-mining in chemistry J Cheminformatics 2011 3 17 10.1186/1758-2946-3-17 PMC3117806 21575201

[B18] SureChem - Chemical Patent Search http://surechem.com/

[B19] Cooke-Fox DI Kirby GH Rayner JD Computer translation of IUPAC systematic organic chemical nomenclature. 1. Introduction and background to a grammar-based approach J Chem Inf Comput Sci 1989 29 101 105 10.1021/ci00062a009

[B20] Cooke-Fox DI Kirby GH Rayner JD Computer translation of IUPAC systematic organic chemical nomenclature. 2. Development of a formal grammar J Chem Inf Comput Sci 1989 29 106 112 10.1021/ci00062a010

[B21] Rocktäschel T Weidlich M Leser U ChemSpot: A Hybrid System for Chemical Named Entity Recognition Bioinformatics 2012 10.1093/bioinformatics/bts183 22500000

[B22] Usie A Alves R Solsona F Vazquez M Valencia A CheNER: chemical named entity recognizer Bioinformatics 2013 10.1093/bioinformatics/btt639 PMC3967102 24227678

[B23] Tang B Feng Y Wang X Wu Y Zhang Y Jiang M Wang J Xu H A comparison of conditional random fields and structured support vector machines for chemical entity recognition in biomedical literature J Cheminform 2015 7 Suppl 1 S8 10.1186/1758-2946-7-S1-S8 PMC4331698 25810779

[B24] Blaschke C Valencia A The frame-based module of the SUISEKI information extraction system IEEE Intell Syst 2002 17 14 20

[B25] Aronson AR Effective mapping of biomedical text to the UMLS Metathesaurus: the MetaMap program Proc AMIA Annu Symp AMIA Symp 2001 17 21 PMC2243666 11825149

[B26] Segura-Bedmar I Martínez P Segura-Bedmar M Drug name recognition and classification in biomedical texts Drug Discov Today 2008 13 816 823 10.1016/j.drudis.2008.06.001 18602492

[B27] Segura-Bedmar I Crespo M de Pablo-Sánchez C Martínez P Resolving anaphoras for the extraction of drug-drug interactions in pharmacological documents BMC Bioinformatics 2010 11 S1 20406499 10.1186/1471-2105-11-S2-S1 PMC3288782

[B28] Segura-Bedmar I Martínez P de Pablo-Sánchez C Extracting drug-drug interactions from biomedical text BMC Bioinformatics 2010 11 S5 10.1186/1471-2105-11-S2-S1 PMC3288782 20406499

[B29] Heerero-Zazo M Segura-Bedmar I Martínez P Declerck T The DDI corpus: an annotated corpus with pharmacological substance and drug-drug interactions Journal of Biomedical Informatics 2013 46 I5 914 920 23906817 10.1016/j.jbi.2013.07.011

[B30] Mallet: A machine learning for language toolkit http://mallet.cs.umass.edu/about.php

[B31] Degtyarenko K de Matos P Ennis M Hastings J Zbinden M McNaught A Alcantara R Darsow M Guedj M Ashburner M ChEBI: a database and ontology for chemical entities of biological interest Nucleic Acids Res 2007 36 D344 D350 10.1093/nar/gkm791 17932057 PMC2238832

[B32] Hastings J de Matos P Dekker A Ennis M Harsha B Kale N Muthukrishnan V Owen G Turner S Williams M Steinbeck C The ChEBI reference database and ontology for biologically relevant chemistry: enhancements for 2013 Nucleic Acids Res 2013 41 D456 D463 10.1093/nar/gks1146 23180789 PMC3531142

[B33] Hettne KM Stierum RH Schuemie MJ Hendriksen PJM Schijvenaars BJA Mulligen EM Kleinjans J Kors JA A dictionary to identify small molecules and drugs in free text Bioinformatics 2009 25 2983 2991 10.1093/bioinformatics/btp535 19759196

[B34] Li Q Cheng T Wang Y Bryant SH PubChem as a public resource for drug discovery Drug Discov Today 2010 15 1052 1057 10.1016/j.drudis.2010.10.003 20970519 PMC3010383

[B35] Choi M Yepes AJ Zobel J Verspoor K NEROC: Named Entity Recognizer of Chemicals Proc Fourth BioCreative Chall Eval Work. Bethesda, Maryland 2013 2 97 104

[B36] Leaman R Wei C-H Lu Z tmChem: a high performance approach for chemical named entity recognition and normalization J Cheminform 2015 7 Suppl 1 S3 10.1186/1758-2946-7-S1-S3 PMC4331693 25810774

[B37] Lowe DM Sayle RA LeadMine: A grammar and dictionary driven approach to chemical entity recognition J Cheminform 2015 7 Suppl 1 S5 10.1186/1758-2946-7-S1-S5 PMC4331695 25810776

[B38] Batista-Navarro RT Rak R Ananiadou S Chemistry-specific Features and Heuristics for Developing a CRF-based Chemical Named Entity Recogniser Proc Fourth BioCreative Chall Eval Work 2013 2 Bethesda, Maryland: Association for Computational Linguistics 55 59

[B39] Huber T Rocktäschel T Weidlich M Thomas P Leser U Extended Feature Set for Chemical Named Entity Recognition and Indexing Proc Fourth BioCreative Chall Eval Work 2013 2 Bethesda, Maryland: Association for Computational Linguistics 88 91

[B40] Khabsa M Giles CL An Ensemble Information Extraction Approach to the BioCreative CHEMDNER Task Proc Fourth BioCreative Chall Eval Work 2013 2 Bethesda, Maryland: Association for Computational Linguistics 105 112

[B41] Akhondi SA Hettne M van der Host E van Mulligen E Kors JA Recognition of chemical entities: combining dictionary-based and grammar-based approaches J Cheminform 2015 7 Suppl 1 S10 10.1186/1758-2946-7-S1-S10 PMC4331686 25810767

[B42] Lana-Serrano S Sanchez-Cisneros D Campillos L Segura-Bedmar I Recognizing Chemical Compounds and Drugs: a Rule-Based Approach Using Semantic Information Proc Fourth BioCreative Chall Eval Work 2013 2 Bethesda, Maryland: Association for Computational Linguistics 121 128

[B43] Yoshioka M Dieb TM Ensemble Approach to Extract Chemical Named Entity by Using Results of Multiple CNER Systems with Different Characteristic Proc Fourth BioCreative Chall Eval Work 2013 2 Bethesda, Maryland: Association for Computational Linguistics 162 167

[B44] Li L Guo R Liu S Zhang P Zheng T Huang D Zhou H Combining Machine Learning with Dictionary Lookup for Chemical Compound and Drug Name Recognition Task Proc Fourth BioCreative Chall Eval Work 2013 2 Bethesda, Maryland: Association for Computational Linguistics 171 177

